# THE READYR PROGRAM FOR WELL-BEING IN DEMENTIA: PILOT STUDY AND SIX-MONTH FOLLOW-UP RESULTS AMONG SPOUSE–PARTNER DYADS

**DOI:** 10.1093/geroni/igad104.1320

**Published:** 2023-12-21

**Authors:** Lyndsey Miller, Joel Steele, Shirin Hiatt, Jeffrey Kaye, Carol Whitlatch

**Affiliations:** Oregon Health & Science University, Portland, Oregon, United States; University of North Dakota, Grand Forks, North Dakota, United States; Oregon Health & Science University, Portland, Oregon, United States; Oregon Health & Science University, Portland, Oregon, United States; Benjamin Rose Institute on Aging, Cleveland, Ohio, United States

## Abstract

The READyR Program assesses and targets everyday routines of care dyads (older adults with cognitive impairment and their co-habiting partners) using in-home sensors and video-conference sessions in order to understand future care needs, and promote greater well-being. This study aimed to evaluate the feasibility, acceptability, and pilot test of efficacy for READyR. Fifteen dyads (96% married) were enrolled in the 6-month study, and randomized to immediately join READyR (n=8 dyads) or to complete READyR after an educational control condition (n=7 dyads). Feasibility was high: 100% of enrolled dyads completed ≥1 session; 80% completed all sessions. Acceptability was also high: responding participants (n=26) reported that READyR helped them express their care values and understand their care needs (92%), that they were comfortable sharing their views (92%) and doing so with their partner present (96%). Hearing and visual impairments were reported to interfere with online sessions for 4 and 6 participants, respectively, but 93% of participants reported that the digital components were easy to use and understand. After receiving the READyR sessions, awareness of future care needs among care partners increased (d = 0.85) and relationship strain for both dyad members decreased (d = 0.58–0.82), when compared to changes among participants in the active control group. No effect was observed on participants’ quality of life, mood, or care-related strain at post-test or at 6 month follow-up. As a remote dyadic intervention, READyR is feasible, acceptable, and may help dyads decrease strain in the care relationship and prepare for future care needs.

